# Salivary Alpha-Amylase Enzyme, Psychological Disorders, and Life Quality in Patients with Recurrent Aphthous Stomatitis

**DOI:** 10.1155/2017/5269856

**Published:** 2017-03-19

**Authors:** Juliana Andrade Cardoso, André Avelino dos Santos Junior, Maria Lucia Tiellet Nunes, Maria Antonia Zancanaro de Figueiredo, Karen Cherubini, Fernanda Gonçalves Salum

**Affiliations:** ^1^Oral Medicine Division, São Lucas Hospital, Pontifical Catholic University of Rio Grande do Sul (PUCRS), Porto Alegre, RS, Brazil; ^2^Cellular and Molecular Biology, Pontifical Catholic University of Rio Grande do Sul (PUCRS), Porto Alegre, RS, Brazil; ^3^School of Psychology, Pontifical Catholic University of Rio Grande do Sul (PUCRS), Porto Alegre, RS, Brazil; ^4^São Lucas Hospital, Pontifical Catholic University of Rio Grande do Sul (PUCRS), Porto Alegre, RS, Brazil

## Abstract

*Objective*. The aim of this study was to evaluate stress, anxiety, and salivary alpha-amylase (SAA) activity in patients with recurrent aphthous stomatitis (RAS). The impact of this disease on the life quality was also evaluated.* Design*. Twenty-two patients with RAS and controls, matched by sex and age, were selected. Stress and anxiety were assessed using Lipp's Inventory of Stress Symptoms and Beck Anxiety Inventory. Life quality was assessed through the World Health Organization Quality of Life-bref (WHOQOL-BREF) and the Oral Health Impact Profile-14 (OHIP-14). Saliva samples were collected in the morning and afternoon and the SAA activity was analyzed by enzymatic kinetic method.* Results*. No significant difference was observed between the groups regarding the SAA activity (*p* = 0.306). Patients with RAS had higher scores of anxiety (*p* = 0.016). The scores of WHOQOL-BREF were significantly lower in patients with RAS. The values obtained through OHIP-14 were significantly higher in these patients (*p* = 0.002).* Conclusion*. RAS negatively affects the life quality. Patients with the disease have higher levels of anxiety, suggesting its association with the etiopathogenesis of RAS.

## 1. Introduction

The recurrent aphthous stomatitis (RAS) is one of the most frequent disorders of the oral mucosa and is characterized by single or multiple and relapsing ulcerations [1]. The lesions are painful and may impair feeding, speech, and oral hygiene, leading to more severe impact on life quality [2]. Its development involves deregulation in mechanisms of oral mucosal response against exogenous or endogenous antigens. There is an imbalance of TCD4+ cells with proliferation of TCD8+ lymphocytes, mediators of cytotoxic reaction [3, 4]. Studies suggest increased Th1 activity relative to Th2 in patients with aphthous stomatitis [5–9]. Several factors have been investigated in the etiology of RAS [10], but the mechanism that triggers the development of lesions remains unknown [11]. Nutritional deficiencies [11–14], hypersensitivity to certain foods [15, 16], local trauma in genetically susceptible patients [10], and psychological disorders [17–21], among others, have been studied. Stress and anxiety seem to be factors associated with the RAS, since higher levels of these psychological disorders have been observed in patients with the disease [17–21]. Gallo et al. [20] suggest that the psychological stress can act as a trigger, increasing the susceptibility of patients predisposed to develop aphthous stomatitis.

The salivary alpha-amylase enzyme (SAA) is produced by the salivary glands and its main function is to initiate the digestion of macromolecules such as carbohydrates [22–24]. Its release is regulated by the sympathetic autonomic nervous system (SANS), whose action is of paramount importance in the psychobiology of stress [25–28]. Studies show that the SAA levels in humans increase under physical and psychological stress [28–30]. For this reason, the SAA has become a marker of stress and anxiety [27], being a fast, painless, and noninvasive tool [25–28, 31] for assessment of the ANS pathological dysregulation in specific clinical and subclinical conditions [27].

The present study was based on a master thesis [32] and aimed at analyzing the salivary alpha-amylase activity, as well as psychological disorders in patients with RAS in attempt to establish possible factors associated with this disease and its relationship to a salivary biomarker. The RAS impact on the life quality was also evaluated.

## 2. Materials and Methods

This clinical, transversal, observational, and controlled study was approved by the Ethics Committee in Research of the Pontifical Catholic University of Rio Grande do Sul (PUCRS Protocol-11/05581). The sample consisted of 52 individuals of both sexes, aged between 19 and 69 years, who were divided into RAS group (22 patients with RAS, who should have had at least three outbreaks of lesions in the last year) and control group (30 patients with no known history of RAS), matched by sex and age. Patients signed an informed consent form.

The study excluded individuals with systemic diseases that could be associated with the development of lesions in the oral mucosa (systemic erythematosus lupus, Behçet's syndrome, Crohn's disease, cyclic neutropenia, or AIDS), who presented a history of malignant neoplasia, chemotherapy or radiotherapy, smokers, users of antidepressants, anxiolytics, corticosteroids, beta-blockers, analgesics containing caffeine, or tetracycline. Patients with periodontal diseases, pregnancy, hypersensitivity to toothpaste and oral mouth rinse solutions, and infectious, erosive, or ulcerative lesions in the oral mucosa as well as those with abnormal blood counts, levels of glucose, iron, folic acid, and vitamin B12 were also excluded from the sample.

The RAS diagnosis was based on the clinical appearance of the lesions and the patients' history according to the criteria established by Chattopadhyay and Shetty [1] and Preeti et al. [33]. Patients with RAS were recruited consecutively in the outpatient clinic of the Oral Medicine Department, Hospital São Lucas-PUCRS, and the control group was selected at the Faculty of Dentistry-PUCRS. During the interview the medical history and medications used by patients were recorded. Individuals with RAS were asked about the disease progression, the frequency of outbreaks, and its association with trigger factors. On the intraoral examination the number of lesions, their location, and clinical form were recorded.

### 2.1. Evaluation of Symptoms of Anxiety and Stress

For the investigation of anxiety and stress the Beck Anxiety Inventory (BAI) and Lipp's Inventory of Stress Symptoms for adults (LISS) were used, respectively. Both psychometric instruments were validated for the Brazilian population and recognized by the Federal Council of Psychology, through a favorable opinion dated 11/06/2003.

### 2.2. Life Quality Assessment

The World Health Organization Quality of Life-bref (WHOQOL-BREF) was used to assess the overall life quality and the Oral Health Impact Profile-14 (OHIP-14) was employed to assess the life quality regarding the oral health of patients. The Portuguese versions of both questionnaires were also validated in Brazil.

### 2.3. Collection of Saliva Samples

The patients' saliva was collected twice in the same day, between 8:00 a.m. and 9:00 a.m. (before breakfast and at least 1 hour after awakening) and between 2:00 p.m. and 5:00 p.m. Disposable flasks with a capacity of 80 ml were used; they were properly identified for each collection period.

Samples were collected during the week, between Monday and Thursday. Patients were advised not to drink alcohol for 24 hours before, eat or drink nor put any substance in the mouth (including not brushing teeth), do physical exercises, and consume caffeine or apply drugs or cosmetics in lips for at least one hour before collecting saliva. Patients should remain seated with their eyes open and deposit the saliva on the flask according to the accumulation in the mouth for long enough so that it would reach the mark of 1.5 ml. The containers were stored in the freezer until the next day, when they were collected by the researcher. The flasks were stored at −20°C.

### 2.4. Analysis of Salivary Alpha-Amylase

The SAA activity was determined by enzymatic kinetic reaction with the Salivary *α*-Amylase Assay Kit (Salimetrics™, 101 Innovation Blvd., Canada-USA), on serial batteries, following the manufacturer's guidelines. The chromogenic substrate 2-chloro-p-nitrophenol linked with maltotriose was used. The enzyme action on this substrate was measured spectrophotometrically at 405 nm.

### 2.5. Statistical Analysis

Variables were initially analyzed using descriptive statistics. The Mann–Whitney test was applied for comparison of SAA activity and scores of inventories BAI and OHIP-14 between groups. Student's *t*-test for independent samples was used to evaluate the WHOQOL-BREF inventory data. The chi-square (*χ*^2^) was used for comparison of presence of stress and anxiety levels between groups. Pearson's correlation coefficient was used to determine the correlation between the SAA activity, anxiety scores, and life quality within the groups. The significance level was *p* ≤ 0.05. The SPSS version 17 (SPSS Inc., Chicago, IL, USA) was used.

## 3. Results

### 3.1. Sample Characterization

In the RAS group, 18 (81.8%) patients were female and four (18.2%) were male. In the control group, 23 (76.7%) patients were female and seven (23.3%) were male. The age of the patients ranged from 18 to 69 years. The mean age of RAS group was 33.8 (±15.7) years and control group was 30.20 (±12.87) years. All patients in the RAS group had minor aphthous, and there were 29 lesions. The affected sites were tongue (31.0%), labial mucosa (31.0%), alveolar mucosa (24.2%), buccal mucosa (6.9%), and mouth floor (6.9%). With respect to trigger factors, 31.8% of patients associated the onset of aphthous stomatitis with stress and 22.7% with acidic foods, 4.5% had this association with anxiety, and 41.0% did not correlate the appearance of lesions with triggering agents. Lesions were perceived from childhood by 50% of patients and from the fourth decade of life by 27.3%, and 22.7% could not specify the time of disease progression. In 72.7% of patients the outbreaks of RAS occurred less than once per month and the frequency was more than once per month in 27.3% of cases.

There was no significant difference in the SAA activity between RAS and control groups in the samples of morning or afternoon (*p* = 0.326) (Table 1).

### 3.2. Anxiety, Stress, and Life Quality

The anxiety scores were significantly higher in the RAS group compared to the control group (*p* = 0.016) (Table 2). When anxiety was categorized as minimum/mild or moderate/severe it was observed that a significantly higher percentage of patients with RAS showed higher levels of this disorder (*p* = 0.046). In the RAS group, 50.0% of subjects had stress while this number was 36.7% in the control group; however, there was no significant difference between them on this variable (*p* = 0.498) (Table 3).

The scores of physical (*p* = 0.026), psychological (*p* = 0.005), social (*p* = 0.001), and environmental (*p* = 0.040) domains of the WHOQOL-BREF inventory were significantly lower in the RAS group (Table 4). Values obtained through the OHIP-14 were significantly higher in patients of this group (*p* = 0.002) (Table 2); that is, the disease exerted negative influence on life quality.

The anxiety and quality of life scores did not correlate with the activity of SAA (data not shown).

## 4. Discussion

Studies that analyze the SAA activity in oral diseases whose etiopathogenesis may be related to stress and anxiety are still scarce and recent in the international literature. Since some authors have suggested the association of psychological disorders with RAS, considering that the SAA is a marker of SANS activation, this study analyzed the enzyme activity in subjects with aphthous stomatitis. We observed considerable variability in the SAA activity, both among patients with RAS and among the controls, with no significant difference between the groups. Similar results were observed by Kim et al. [34] when investigating, among other markers, the activity of SAA in patients with burning mouth syndrome. The authors found no difference in enzyme levels between patients with the syndrome and controls nor significant correlation of SAA with psychological symptoms such as anxiety and depression. On the other hand, Sánchez et al. [35] observed elevation of this enzyme activity in patients with periodontal disease; however, they suggest that the metabolic stress caused by the inflammatory process is responsible for this finding.

The literature reports increased levels of SAA under various conditions of physical and psychological stress [28–30]. Overall, the studies evaluate the activity of this enzyme in response to acute stress, that is, immediately after stressful situations or tasks. In these cases, there is a significant increase in the SAA activity as an immediate response to the stressor agent [36–40]. This reaction occurs due to the SANS activation, which through the release of catecholamines causes changes in the physiological state [40]. In the salivary glands, the sympathetic innervation causes vasoconstriction, decreased salivary flow, and increased protein and inorganic compounds, mainly alpha-amylase [41]. This is a short-lived response, owing to reflex parasympathetic activation [40]. In this study, patients were evaluated and their saliva was collected within 72 h after presentation of clinical signs of aphthous stomatitis, which may explain the absence of differences in enzymatic levels. Therefore, the SAA was not shown to be a salivary marker associated with RAS.

The combination of stress and anxiety in the RAS etiology has been suggested in the literature [17, 19], since these disorders modify and promote deregulation of immune functions, with an imbalance of Th1/Th2 cytokines [42]. In this study, scores and anxiety levels were significantly higher in subjects with RAS when compared to controls. The percentage of patients with stress was also higher in the RAS group but with no significant difference compared to the control. The lack of difference between groups can be explained by the sample size and because the stress has been assessed as a dichotomous variable. Similar results were found by McCartan et al. [43], Soto-Araya et al. [17], Albanidou-Farmaki et al. [19], and Gallo et al. [20] when assessing anxiety in patients with RAS. However, it remains controversial whether these psychological alterations are associated with etiology or develop as a consequence of pain caused by the RAS. For Picek et al. [44], psychological disorders do not precede the development of lesions and patients are more anxious due to the discomfort caused by them.

Regarding the life quality, the scores of the four domains of the WHOQOL-BREF Inventory, which evaluates the overall life quality, were significantly lower in RAS group. The values obtained from the OHIP-14 instrument, which assesses the impact of oral conditions on life quality, were significantly higher in patients of this group. The lower the WHOQOL-BREF scores, the worse the life quality of patients. In contrast, higher scores of OHIP-14 indicate greater impact of oral health on life quality. Therefore, the results of this study demonstrate that the RAS has a negative impact on both overall life quality and oral health. Similar results were observed by Mumcu et al. [18] and Krisdapong et al. [2], once this disease causes pain, discomfort, difficulty in feeding, and speech, harming the emotional stability of patients.

In this study, the RAS was more prevalent in female patients, the lesions were minor aphthous, and in 50% of cases the disease started in childhood. These results corroborate the literature, since the RAS affects mainly women [21, 45] and minor aphthous are the most prevalent, with its initial development in childhood [1, 10, 15]. The lesions were observed mainly in the ventral edges of the tongue and labial mucosa, confirming their preference for nonkeratinized mucosa [1, 10, 11, 17, 20].

The RAS is a complex, multifactorial disease, and although not a morbid entity, it negatively affects the life quality of individuals. We can conclude that patients with the disease have higher levels of anxiety, suggesting its association with the etiopathogenesis of RAS. Moreover, despite being a biomarker of stress, the SAA release oscillates rapidly and may be influenced by several factors, not being related to stress and anxiety in patients with RAS. Further studies are needed to elucidate the role of these psychological disorders in the RAS etiopathogenesis.

## Figures and Tables

**Table 1:** Activity of the salivary alpha-amylase enzyme (U/ml) in the RAS and control groups.

Salivary alpha-amylase (U/ml)	RAS group *n* = 22	Control group *n* = 30	*p*
	Median (P25–P75)	Median (P25–P75)	
Morning samples	42.72 (25.18–75.55)	45.50 (35.68–97.47)	*0.326*
Afternoon samples	83.47 (54.89–136.26)	74.85 (50.42–124.23)	*0.326*

Mann–Whitney's test is significant at *p* ≤ 0.05.

(P25–P75) = 25–75th percentiles.

**Table 2:** Scores of the Beck Anxiety Inventory (BAI) and the Oral Health Impact Profile-14 (OHIP-14) in the RAS and control groups.

	RAS group *n* = 22	Control group *n* = 30	*p*
	Median (P25–P75)	Median (P25–P75)	
BAI	10.50 (5.00–28.25)	5.50 (1.00–10.00)	*0.016*
OHIP-14	8.35 (1.17–13.93)	2.75 (0.00–4.53)	*0.002*

Mann–Whitney's test is significant at *p* ≤ 0.05.

**Table 3:** Prevalence of stress obtained through Lipp's Inventory of Stress Symptoms for Adults (LISS) in the RAS and control groups.

	RAS group *n* = 22	Control group *n* = 30	*p*
	Yes	Yes	
Stress	11 (50.0%)	11 (36.7%)	*0.498*

Chi-square test, significant at *p* ≤ 0.05.

**Table 4:** Scores of physical, psychological, social, and environmental domains of the World Health Organization Quality of Life-bref (WHOQOL-BREF) inventory in the RAS and control groups.

WHOQOL-BREF domains	RAS group *n* = 22	Control group *n* = 30	*p*
	Mean ± SD	Mean ± SD	
Physical	70.1 ± 13.5	78.5 ± 12.5	*0.026*
Psychological	66.9 ± 13.8	76.9 ± 8.8	*0.005*
Social	65.7 ± 19.2	82.5 ± 12.6	*0.001*
Environmental	61.1 ± 13.9	68.7 ± 11.9	*0.040*

Student's *t*-test for independent samples, significant at *p* ≤ 0.05.
